# Mean platelet volume as a predictive biomarker for in-hospital mortality in patients receiving invasive mechanical ventilation

**DOI:** 10.1186/s12890-022-02155-z

**Published:** 2022-09-18

**Authors:** Yingying Zheng, Zujin Luo, Zhixin Cao

**Affiliations:** Department of Respiratory and Critical Care Medicine, Beijing Institute of Respiratory Medicine and Beijing Chao-Yang Hospital, Capital Medical University, Beijing, China

**Keywords:** Mean platelet volume, Invasive mechanical ventilation, In-hospital mortality

## Abstract

**Background:**

Although mean platelet volume (MPV) has been reported to be associated with poor prognosis of various critical illness, the relationship between MPV and in-hospital mortality among patients undergoing invasive mechanical ventilation (IMV) is unclear.

**Methods:**

A retrospective observational study including patients receiving IMV was conducted from January, 2014 to January, 2019. The patients were divided into two groups by MPV cutoff value. The receiver operating characteristics curve was used to evaluate the predictive ability of MPV for in-hospital mortality. Univariate and multivariate Cox regression analysis were conducted to analyze the value of MPV for predicting in-hospital mortality. Kaplan–Meier cumulative incidence curve was employed to observe the incidence of in-hospital mortality.

**Results:**

A total of 274 patients were enrolled in the study, and 42 patients (15.3%) died in hospital. MPV > 11.4 fl was a valuable predictor for in-hospital mortality (AUC0.848; 95%CI, 0.800–0.889) with sensitivity 66.7%, and specificity = 86.21%. MPV > 11.4 fl was an independent risk factor for in-hospital mortality (adjusted HR 2.640, 95%CI, 1.208–5.767, *P* = 0.015). Compared to the group of MPV ≤ 11.4 fl, patients with MPV > 11.4 fl had increased mortality (log-rank test = 40.35, HR = 8.723, *P* < 0.0001). The relationship between MPV and in-hospital mortality was stronger in female patients than in male patients.

**Conclusion:**

MPV > 11.4 fl is a more useful marker for predicting in-hospital mortality among critically ill patients receiving IMV, especially in female patients. Attention to the MPV marker is simple and profitable with immediate applicability in daily clinical practice.

## Introduction

Mechanical ventilation is frequently used for life support for patients experiencing respiratory failure or undergoing general anesthesia in critical illness. These patients are at risk for a number of complications related to both their underlying disease state and the mechanical ventilation itself [1]. Some studies evaluating patients requiring mechanical ventilation have reported mortality rates from 12 to 40% [2–9] according to different diseases. Therefore, early predictions of mortality and intervention are of great importance for improving the prognosis of patients. Acute Physiology and Chronic Health Evaluation II (APACHE II) score, solid tumor, severe sepsis/septic shock, acute lung injury/acute respiratory distress syndrome, and acute kidney injury have been reported as predictors of hospital mortality [9]. However, the relationship between inflammation markers and hospital mortality among those adult patients receiving IMV were lacking. Hence, it is critical to explore the predictive ability of inflammation markers for hospital mortality for those patients.

It has been reported that platelet has increasing focus for its role in inflammation and immunity [10]. Mean platelet volume (MPV), a routinely measured marker in clinical practice, is a simple, easy, available, and accurate indicator of platelet size and function [11]. Numerous studies have demonstrated that MPV levels or the change of MPV were correlated with serious compilation or poor prognosis for ICU patients [12, 13], sepsis [14, 15], influenza pneumonia [16], acute pulmonary embolism [17].In addition, MPV also has been shown to be a valuable prognostic marker for patients following acute abdominal surgery [18], chronic obstructive pulmonary disease (COPD) patients [19], coronary artery disease [20] and Type 2 Diabetes Mellitus [21].

Critically ill patients experiencing invasive mechanical ventilation(IMV) are generally in a high state of inflammation, strong stress, and immunity response [22], which may be related to both their underlying disease state, endotracheal intubation and the mechanical ventilation itself [1]. Nonsurvivors may suffer more inflammatory response and stress. However, there were few studies focusing on the inflammation markers and in-hospital mortality among critically ill adult patients receiving IMV.

Accordingly, we assumed that higher MPV could be related with higher in-hospital mortality. And we evaluated the effectiveness of MPV for predicting hospital mortality in patients receiving IMV.

## Methods

### Study population

This retrospective, observational, single center study included patients receiving IMV not less than 24 h admitted to the ICU of Beijing Chao-Yang Hospital from January, 2014 to January, 2019. The following patients were excluded: Age < 18 years; pregnancy; tracheotomy or other upper airway disorders; mechanically ventilated less than 24 h; neuromuscular disease; decision to abandon or limit active treatment; incomplete data [23].

This study (NO.2020-KE-94) was approved by the Ethics Committee of the Beijing Chao-Yang Hospital, Capital Medical University. The requirement for informed consent was waived by the Ethics Committee of the Beijing Chao-Yang Hospital, Capital Medical University because of the retrospective nature of the study. All procedures were in accordance with Helsinki Declaration.

### Data collection

Patients’ demographic and baseline characteristics were recorded within 24 h after intubation, including age, sex, body mass index (BMI), acute physiology and chronic health evaluation II (APACHE II) score, basic vital signs, arterial blood gas and laboratory data. The following were also recorded: comorbidities and acute causes of IMV, IMV duration, complication, length of stay (LOS) in ICU, LOS in hospital, and in-hospital mortality. Albumin (ALB), alanine aminotransferase (ALT), aspartate aminotransferase (AST), blood creatinine, complete blood cell counts and CRP within 24 h after intubation were measured. Complete blood cell counts were measured by an automatic blood analyzer. CRP concentrations were measured using immunoscatter turbidimetry by Goldsite Aristo (Goldsite, Ltd., China), and the normal value of CRP ranges from 0 to 5 mg/L [23]. Acute kidney injury(AKI), defined according to established criteria [24]; septic shock, defined according to established criteria [25]. Gastrointestinal bleeding was defined as any clinically suspected or documented of bleeding from the gastrointestinal tract as indicated with a fall in hemoglobin level and the appearance of melena, hematochezia, haematemesis or stool tested positive for occult blood [26]. Acute liver dysfunction was defined as abnormality of the AST and ALT. Acute coronary syndrome included the event of acute ST elevation or nonST elevation myocardial infarction or unstable recurrent angina as indicated by the typical symptom with abnormal ECG or echocardiography or myocardial enzyme elevation. Acute cerebrovascular disease included haemorrhagic stroke, ischaemic stroke and transient ischaemic attack which were diagnosed or suspected by neurologist according to symptoms and computerized tomography of brain.

### Clinical outcome

The primary outcome was in-hospital mortality rate, which was defined as deaths occurring during hospitalization. Survivors were discharged to homes or transferred to another hospital.

### Statistics

Continuous variables were expressed as mean ± standard deviation (SD) for normality distribution or median (25th-75th percentile) for nonnormally distributed data.

The Kolmogorov–Smirnov test was used for the normality distribution. Student’s *t* test was employed for normally distributed data. The Mann–Whitney U-test was used for nonnormally distributed data. Categorical data was expressed as frequency and percentages, and Chi square (*χ*2) test was performed. Spearman correlations were used for correlation analysis between MPV and other variables. A univariate Cox regression analysis was constructed to explore the association between patients’ characteristics and in-hospital mortality. The multivariate Cox regression with the enter method was also employed to examine variables significantly associated with in-hospital mortality in the univariate analysis, including covariates: APACHE II score, MPV, PaO2/FIO2, vasopressor use, CRRT, CKD, AKI, and septic shock, which resulted in adjusted hazard ratios (HR, 95%CI). In Cox models, time at risk was from study entry until death during hospitalization, discharge, or transfer. Furthermore, the receiver operating characteristic (ROC) analysis was used to evaluate the sensitivity and specificity of MPV for predicting in-hospital mortality and to determine the optimum cutoff value. Cumulative survival curves were conducted by Kaplan–Meier method and differences between groups were evaluated by log-rank test. All analyses were two-tailed, and a probability value (p value) less than 0.05 was considered statistically significant. All data were done using SPSS (Statistical Package for the Social Science; SPSS Inc., Chicago, IL, USA) version 22 for Microsoft Windows.

## Results

### Patients’ characteristics and outcomes

**Fig. 1:**
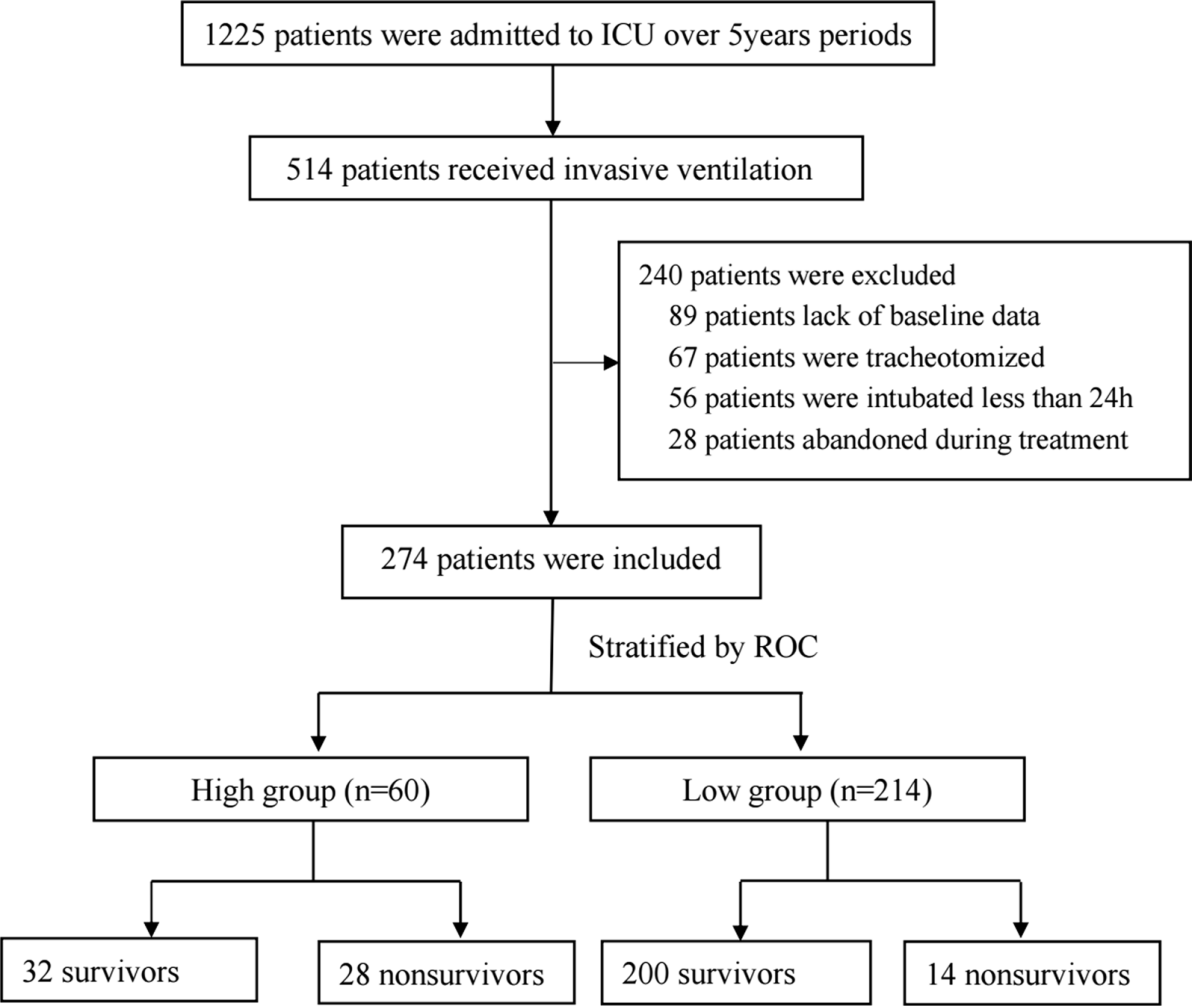
Flow chart of patient selection and outcome. Note: high group: MPV > 11.4 fl; low group, MPV ≤ 11.4 fl; ROC, receiver operating characteristic curve; ICU, intensive care unit

A total of 274 patients were enrolled in the study, as shown in Fig. 1. Of these, 42 patients (15.3%) died in hospital. According to the optimal cutoff value of MPV predicting in-hospital mortality, baseline characteristics and outcomes were summarized in Table 1. Compared with patients with MPV ≤ 11.4 fl, patients with MPV > 11.4 fl had higher APACHE II score, increased frequency of patients with congestive heart failure, and immunosuppression, longer ICU LOS, much more complications including septic shock, AKI, acute liver dysfunction, gastrointestinal bleeding, and acute coronary syndrome, higher frequency treatment of vasopressor use and CRRT. Moreover, patients with MPV > 11.4 fl showed lower PaO2/FIO2, lower platelet count, higher lactate, higher PDW, higher larger platelet ratio, and higher CRP level. The hospital mortality rate was higher in patients with MPV > 11.4 fl than those with MPV ≤ 11.4 fl (46.67% vs 6.54%, *P* = 0.000).

**Table 1:** Baseline characteristics based on the MPV cutoff value

Characteristics	MPV > 11.4 fl (n = 60)	MPV ≤ 11.4 fl (n = 214)	*P*
Age, year (mean ± SD)	72.75 ± 11.24	70.77 ± 13.52	0.300
Male, n (%)	37 (61.67)	123 (53.02)	0.561
BMI, kg/m^2^ (mean ± SD)	24.02 ± 3.42	24.31 ± 4.71	0.597
APACHE II score, (mean ± SD)	21.02 ± 6.67	18.44 ± 6.96	0.007
IMV duration, days (median, 25th–75th percentiles)	7 (4–16)	4 (1–9.25)	0.053
ICU LOS, days (median, 25th–75th percentiles)	17 (10–32)	10 (3–20)	0.000
H LOS, days (median, 25th–75th percentiles)	21 (15.25–40.75)	19 (13–28)	0.069
*Cause of mechanical ventilation, n (%)*			
Exacerbation of chronic respiratory disorders	15 (25)	37 (17.29)	0.178
Pneumonia	18 (30)	62 (28.97)	0.887
Sepsis	12 (20)	43 (20.09)	0.987
Postoperation	8 (13.33)	52 (24.30)	0.070
Congestive heart failure	4 (6.67)	3 (1.40)	0.040
Neurological disease	2 (3.33)	12 (5.61)	0.741
others	1 (1.67)	5 (2.34)	1.000
*Comorbidity disease, n (%)*			
Hypertension	36 (60)	114 (53.27)	0.335
Diabetes Mellitus	23 (38.33)	54 (25.23)	0.050
Chronic heart disorders	21 (35)	64 (29.9)	0.451
Chronic respiratory disorders	19 (31.67)	61 (28.5)	0.634
Malignancy	14 (23.33)	49 (22.90)	0.943
Chronic kidney disease	11 (18.33)	22 (10.28)	0.090
Cerebrovascular disease	10 (16.67)	33 (15.42)	0.815
Immunosuppression	3 (5)	1 (0.46)	0.034
*Clinical outcome, n (%)*			
Septic shock	25 (41.67)	41 (19.16)	0.000
Acute renal injury	29 (48.33)	41 (19.16)	0.000
Gastrointestinal bleeding	18 (30)	36 (16.82)	0.023
Acute liver dysfunction	26 (43.33)	61 (28.50)	0.029
Acute coronary syndrome	8 (13.33)	12 (5.61)	0.042
Acute cerebrovascular disease	3 (5)	8 (3.74)	0.710
Vasopressor use	19 (31.67)	39 (18.22)	0.024
CRRT	3 (5)	11 (5.14)	1.000
Sedation	46 (76.67)	146 (68.22)	0.207
Hospital mortality	28 (46.67)	14 (6.54)	0.000
*Laboratory findings*			
RR, breaths/min	21.10 ± 5.252	19.83 ± 4.712	0.074
SpO_2_, %	98 (96.25–100)	99 (97.75–100)	0.057
HR, beats/min	87.70 ± 15.358	87.44 ± 14.794	0.907
SPB, mmHg	130.38 ± 18.94	129.50 ± 21.98	0.778
PH	7.45 (7.42–7.48)	7.45 (7.42–7.48)	0.718
PaCO_2_, mmHg	54.5 (45.95–71)	50 (44.3–60.7)	0.054
PaO_2_, mmHg	89 (78.65–95)	89 (80–101.5)	0.148
PaO_2_/FiO_2_, mmHg	153.87 (130.26–172.86)	161.82 (138.33–187.38)	0.035
Lactate, mmol/L	1.6 (1.2–2.5)	1.3 (1.00–1.7)	0.002
Hemoglobin, g/L	93.13 ± 24.90	99.10 ± 21.73	0.070
Albumin, g/L	26.08 ± 6.12	27.53 ± 6.65	0.130
Creatinine	88.85 (55.43–125.30)	74 (57.4–117.3)	0.363
ALT, IU/L	23 (16.63–32.5)	19.15 (13–34.65)	0.100
AST, IU/L	32.55 (21.18–53.75)	26.9 (18.5–43.05)	0.095
Leukocyte count, × 10^9^/L	10.25 (8.25–12.05)	9.7 (6.9–12.65)	0.546
Neutrophils count, × 109/L	7.52 (5.82–9.84)	8.29 (5.8–11.74)	0.380
Lymphocyte count, × 10^9^/L	0.73 (0.5–1.07)	0.79 (0.53–1.29)	0.303
Platelet count, × 10^9^/L	148 (107.5–202.75)	184 (134.75–254.5)	0.003
PDW, %	14.45 (11.53–16.48)	12 (10.5–12.925)	0.000
Platelet large cell ratio, %	34.02 ± 9.90	27.44 ± 7.74	0.000
CRP, mg/L	70.5 (34.5–109.5)	44.5 (18.75–84.0)	0.002

Continuous variables were presented as median (25th-75th percentile) or (mean ± SD). Categorical variables were presented as numbers (n) and percentages (%)

*BMI* body mass index; *APACHE II* acute physiology and chronic health evaluation II; *IMV* invasive mechanical ventilation; *LOS* length of stay; *RR* respiratory rate; *HR* heart rate; *SBP* systolic blood pressure; *SPO*_2_ peripheral oxygen saturation; *PaCO*_2_ arterial carbon dioxide tension; *PaO*_2_ arterial oxygen tension; *FiO*_2_ fraction of inspired oxygen; *MPV* mean platelet volume; *PDW* platelet distribution width; *CRP* C-reactive protein; *CRRT* continuous renal replacement therapy; *ALT* alanine aminotransferase; *AST* aspartate aminotransferase

### Relationship between MPV and other variables

**Fig. 2:**
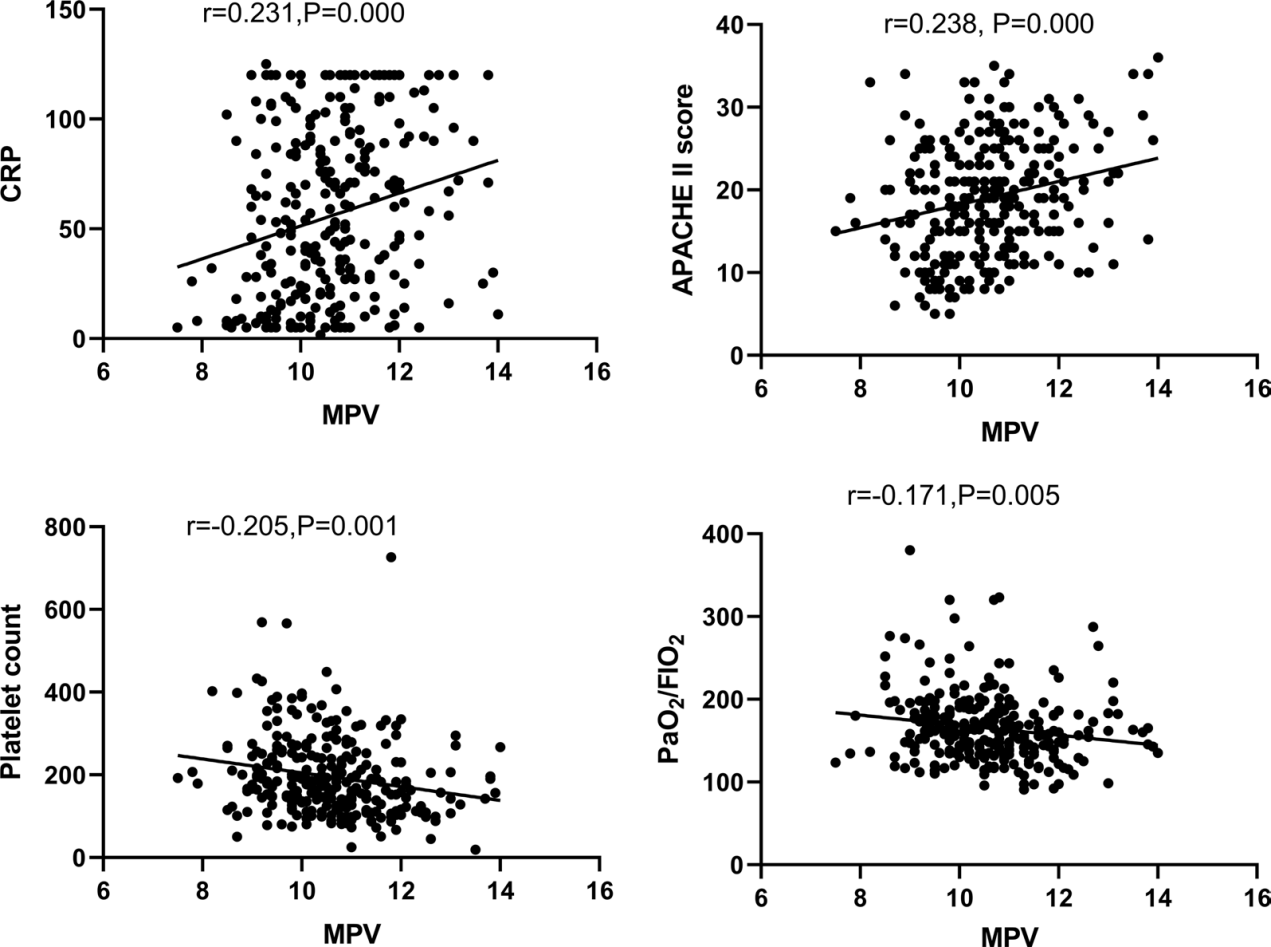
Correlations between MPV and other variables. Abbreviate: MPV, mean platelet volume; APACHE II, acute physiology and chronic health evaluation II; PaO_2_, arterial oxygen tension; FiO_2_, fraction of inspired oxygen; CRP, C-reactive protein

By the correlation analysis as shown in Fig. 2, MPV was positively related with CRP (r = 0.231, *P* = 0.000), APACHE II score (r = 0.238, *P* = 0.000), while it was negatively correlated with platelet count (r =  − 0.205, *P* = 0.001) and PaO_2_/FIO_2_ (r =  − 0.171, *P* = 0.005).

### Risk factors for in-hospital mortality

The possible risk factors for higher in-hospital mortality were shown in Table 2. After adjusting for covariates (P < 0.05 in univariate Cox regression), the multivariate Cox regression suggested that MPV, PaO_2_/FIO_2_, CRP and CRRT were associated with in-hospital mortality (*P* = 0.001, *P* = 0.015, *P* = 0.008 and *P* = 0.005 respectively).

**Table 2:** Independent predictors of in-hospital mortality by univariate and multivariate Cox regression analysis

Variables	HR (95%CI)	*P*
*Univariate Cox analysis*		
APACHE II score	1.046 (1.001–1.092)	0.043
Albumin	1.016 (0.963–1.072)	0.561
Creatinine	1.001 (1.000–1.002)	0.214
MPV	1.712 (1.358–2.159)	0.000
Leukocyte	0.982 (0.908–0.993)	0.657
CRP	1.011 (1.003–1.020)	0.011
Vasopressor use	2.087 (1.115–3.908)	0.021
CRRT	3.134 (1.193–8.232)	0.003
CKD	2.052 (1.017–4.140)	0.045
Septic shock	2.538 (1.371–4.697)	0.003
AKI	1.949 (1.051–3.612)	0.034
PaO_2_/FIO_2_	0.982 (0.971–0.993)	0.002
Age	1.028 (1.000–1.057)	0.052
Lactate	1.080 (0.953–1.223)	0.228
sex	0.849 (0.447–1.613)	0.849
Multivariate Cox* analysis		
MPV	1.741 (1.267–2.393)	0.001
PaO_2_/FIO_2_	0.984 (0.972–0.997)	0.015
CRP	1.013 (1.003–1.022)	0.008
CRRT	5.455 (1.676–17.759)	0.005

*Covariates included in multivariate analysis: APACHE II score, MPV, CRP, PaO2/FIO2, vasopressor use, CRRT, CKD, AKI, septic shock

*APACHE II* acute physiology and chronic health evaluation II; *MPV* mean platelet volume; *CRP* C-reactive protein; *CRRT* continuous renal replacement therapy; *CKD* chronic kidney disease; *AKI* acute kidney injury; *PaO*_2_ arterial oxygen tension; *FiO*_2_ fraction of inspired oxygen;

### Predicting ability of MPV for in-hospital mortality

**Fig. 3:**
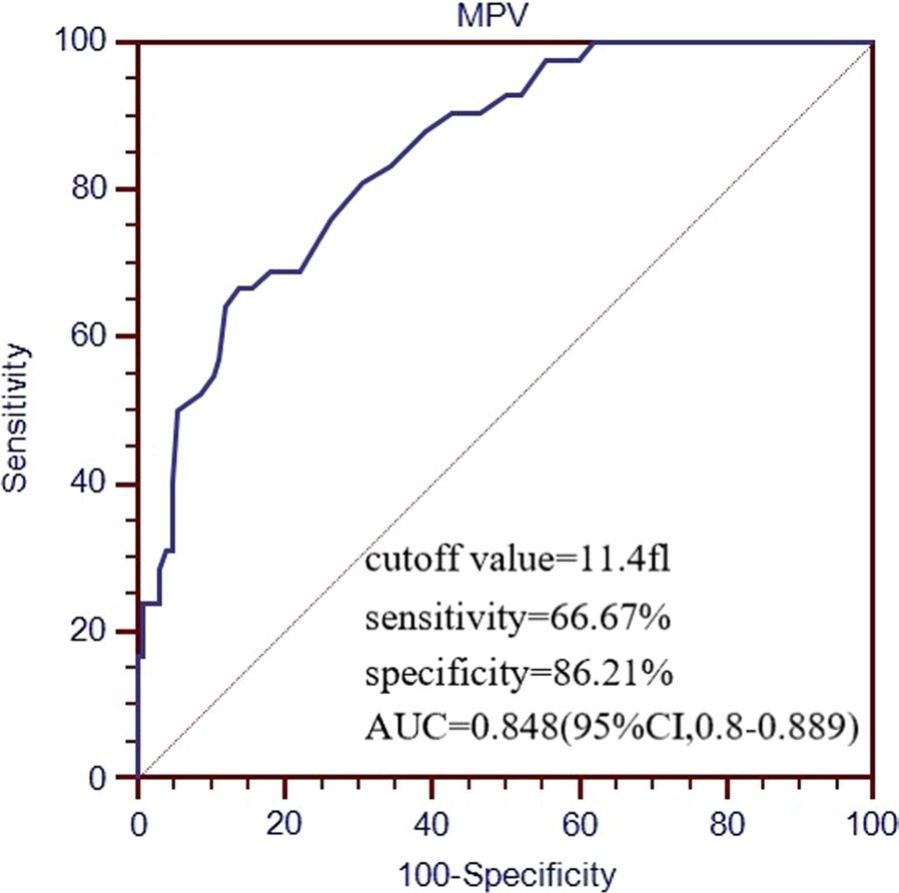
ROC curves of MVP for predicting in-hospital mortality. Abbreviate: MPV, mean platelet volume; ROC, receiver operating characteristic; AUC, area under the curve

As shown by ROC curves (Fig. 3), the cutoff value of MPV for predicting in-hospital mortality was 11.4 fl with a sensitivity of 66.67%, a specificity of 86.21% and diagnostic accuracy of 83.21%, and had a moderate power for predicting in-hospital mortality (AUC 0.848; 95%CI, 0.800–0.889, *P* < 0.0001).

### MPV associated with in-hospital mortality

**Fig. 4:**
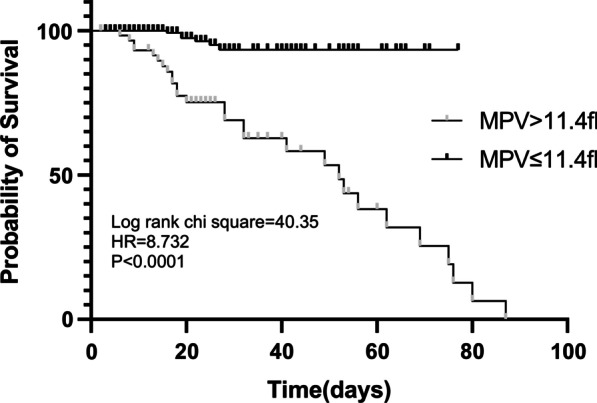
Kaplan–Meier survival curve according to the cutoff value of MPV for in-hospital mortality. MPV, mean platelet volume

According to Kaplan–Meier survival curves for the MPV level, patients with MPV > 11.4 fl had clearly higher mortality than those with MPV ≤ 11.4 fl (log-rank chi square test = 40.35, HR = 8.723, *P* < 0.0001) (Fig. 4).

Associations of MPV with in-hospital mortality were shown in Table 3. After adjusting for covariates, Cox regression analysis indicated that patients with MPV > 11.4 fl was significantly related to higher in-hospital mortality. In model 3 with the maximum covariates including age, sex, APACHE II score, PaO2/FIO2,vasopressor use, CRRT, CKD, AKI, septic shock, albumin, creatinine, leukocyte, and CRP, adjusted HR for in-hospital mortality was 2.640 (95%CI, 1.208–5.767,P = 0.015).

**Table 3:** Relationship between MPV level and in-hospital mortality

In-hospital mortality	MPV > 11.4 fl group	
	HR (95%CI)	*P*
Unadjusted	4.30 (2.223–8.317)	0.000
Model 1	4.245 (2.18–8.266)	0.000
Model 2	3.996 (1.862–8.578)	0.000
Model 3	2.640 (1.208–5.767)	0.015

Reference group is MPV ≤ 11.4 fl. Model 1: age and sex; Model 2: model 1 plus APACHE II score, PaO2/FIO2, vasopressor use, CRRT, CKD, AKI, septic shock; Model 3: model 2 plus albumin, creatinine, leukocyte, and CRP

*MPV* mean platelet volume; *APACHE II* acute physiology and chronic health evaluation II; *CRP* C-reactive protein; *CRRT* continuous renal replacement therapy; *CKD* chronic kidney disease; *AKI* acute kidney injury; *PaO*_2_ arterial oxygen tension; *FiO*_2_ fraction of inspired oxygen; *HR* hazard ratio; *CI* confidence interval

### Relationship between mortality and MPV in the sex subgroup

**Fig. 5:**
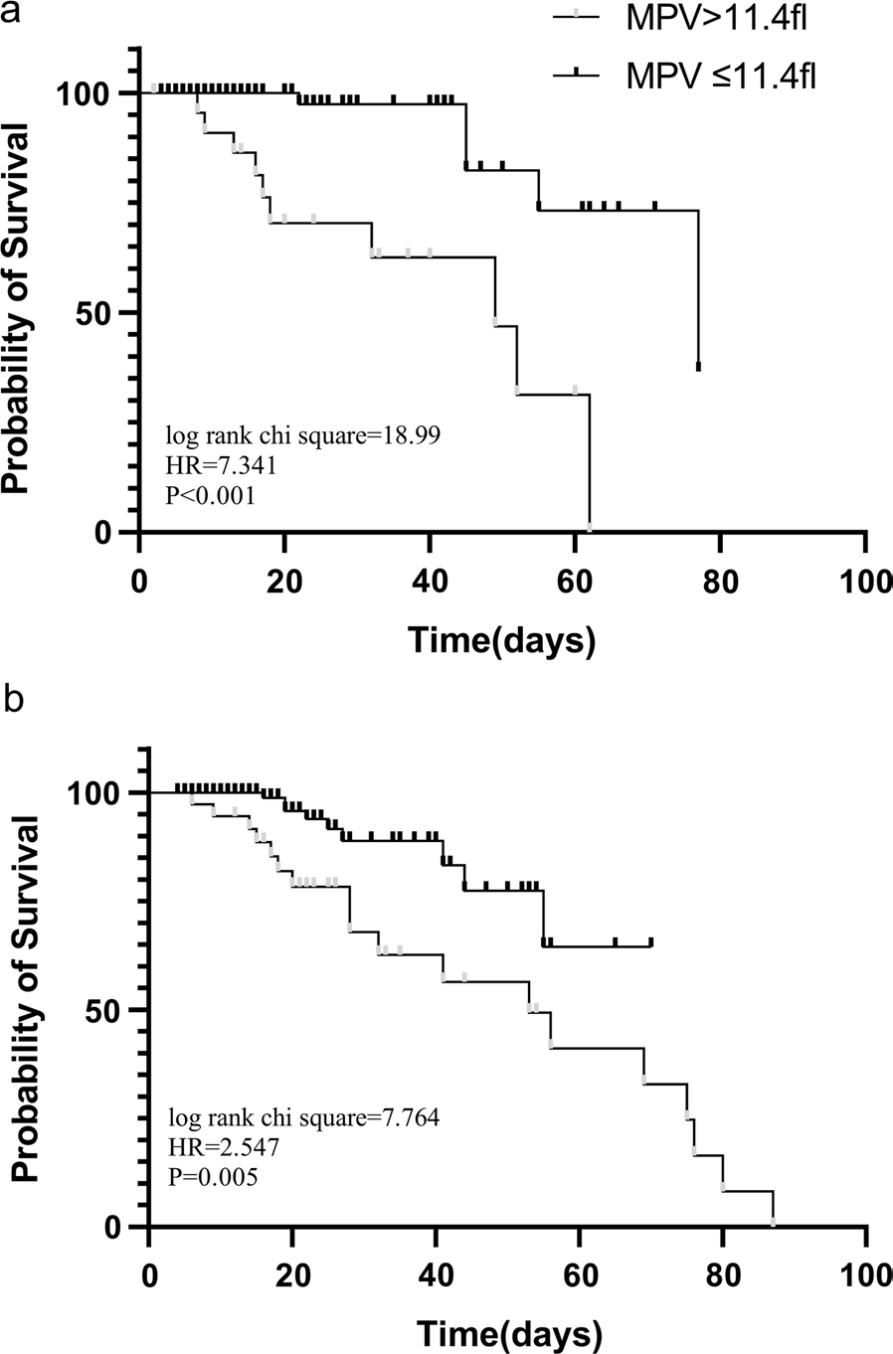
Kaplan–Meier survival curve according to the cutoff value of MPV for in-hospital mortality in female subgroup (**a**) and male subgroup (**b**). MPV, mean platelet volume

A subgroup analysis by sex was conducted using Cox model after adjusting covariates including APACHE II score, CRP, PaO2/FIO2, vasopressor use, CRRT, CKD, AKI, septic shock. In the subgroup analysis, the relationship between MPV > 11.4 fl and in-hospital mortality seemed to be stronger in female subgroup (HR, 14.265;95%CI: 2.323–87.579; *P* = 0.004) than that in male subgroup (HR, 2.905;95%CI:1.042–8.098; *P* = 0.042) (Table 4). The Kaplan–Meier curve also suggested that the MPV > 11.4 fl had a greater impact on in-hospital mortality in female patients than in male patients (Fig. 5).

**Table 4:** Relationship between in-hospital mortality and MPV cutoff by sex

In-hospital mortality	Female		Male	
	HR (95%CI)	*P*	HR (95%CI)	*P*
MPV > 11.4 fl	14.265 (2.323–87.579)	0.004	2.905 (1.042–8.098)	0.042

Covariates included in Cox model: APACHE II score, CRP, PaO2/FIO2, vasopressor use, CRRT, CKD, AKI, septic shock

*MPV* mean platelet volume; *APACHE II* acute physiology and chronic health evaluation II; *CRP* C-reactive protein; *CRRT* continuous renal replacement therapy; *CKD* chronic kidney disease; *AKI* acute kidney injury; *PaO*_2_ arterial oxygen tension; *FiO*_2_ fraction of inspired oxygen; *HR* hazard ratio; *CI* confidence interval

## Discussion

In this research, we indicated MPV > 11.4 fl within 24 h after IMV was positively associated with in-hospital mortality among patients experiencing IMV. MPV > 11.4 fl with the AUC of ROC (0.848; 95%CI, 0.800–0.889) had a moderate predictive ability for in-hospital mortality and MPV > 11.4 fl was an independent predictor for in-hospital mortality. The HR of MPV > 11.4 fl for predicting mortality was 2.642 after adjusting for potential confounders, suggesting that the mortality risk of patients with MPV > 11.4 fl is much higher than patients with MPV ≤ 11.4 fl. The relationship between MPV > 11.4 fl and in-hospital mortality was stronger in female patients than in male patients.

Some studies had reported the similar results. A study suggested that MPV cutoff > 9,45fL at day 1, > 8,95fL at day 2 and > 8, 85fL at day 3 were independent predictor factors of mortality in sepsis [27]. Chen reported that MPV > 10.5 fl were associated with in-hospital mortality in severe pneumonia patients [28]. A retrospective study also found that the elevated MPV was related to poor outcome [29], however they did not find the association between the MPV at admission and in-hospital mortality in ICU patients. In our study, we focused on patients with IMV, and also find MPV > 11.4 fl was associated with in hospital mortality. The cutoff values were different, which may due to the difference in study population, severity of diseases, baseline characteristics, and even adjusted confounders.

The underly mechanisms of higher MPV with poor prognosis in patients with IMV remain hazy. The findings could be elaborated by several possible reasons as below.

Patients with mechanical ventilations are often critically ill and undergo a more serious status and stronger inflammatory response [30, 31]. Endotracheal intubation and mechanical ventilation can enhance this inflammatory reaction and stress. Moreover, nonsurvivors may experience more local and systemic inflammatory responses and stress. Hence, severe inflammatory response and stress were the possible mechanisms for higher MPV in nonsurvivors, which were induced by underlying disease, endotracheal intubation, and mechanical ventilation.

Some studies suggested that serious inflammation could induce a systematic response with the release of thrombopoietin and lots of proinflammatory cytokines, mainly IL-6 and other substances that stimulate platelet activation and the massive production of young platelets in the blood circulation [27, 32, 33], but these large platelets function poorly competent, inducing thrombogenic activity and adverse clinical outcomes [32]. In the current study, patients with MPV > 11.4 fl had higher APACHE II score, longer ICU LOS, more complications including shock, AKI, acute liver dysfunction, gastrointestinal bleeding, and acute coronary syndrome, higher frequency treatment of vasopressor use and CRRT. Moreover, patients with MPV > 11.4 fl showed lower PaO2/FIO2, higher lactate, and higher CRP level, suggesting that those patients suffer more serious status and show more intense inflammatory response. In addition, a previous study had reported this platelet count was inversely related with MPV [34], in this study patients with MPV > 11.4 fl had a lower platelet count than that in patients with MPV ≤ 11.4 fl. MPV was also negatively associated with platelet count among these patients. We inferred that thrombocyte consumption caused by severe inflammation could result in higher MPV. Besides that, some studies reported that hypoxemia may enhance platelet consumption and promote bone marrow activation. Our study showed that nonsurvivors had much lower PaO2/FIO2, which could be another explanation for the increased MPV levels in nonsurvivors. In all, these factors could result in a higher MPV in nonsurvivors. According, we believe that MPV might be a more useful predicting factor for in-hospital mortality.

Besides that, we also found that the relationship between higher MPV and in-hospital mortality was much stronger in female patients than in male patients, which indicating that MPV was more likely to reflect in-hospital mortality in female patients. This was in line with previous study [28], reporting that MPV was associated with mortality among severe pneumonia female patients. Although it has been reported that there were differences in platelet function between male and female mice [35], the mechanism of the relationship was unclever. Female have higher platelet responsiveness [36], platelet counts [37], and had a higher propensity to activate integrin αIIbβ3, to degranulate and expose P-selectin, and to form platelet–leukocyte aggregates [38]. Women platelets have an intrinsic higher propensity to be activated by inflammatory stimuli compared to men [39]. The sexual dimorphism affects both the pro-aggregating and the immune-modulatory function of platelets [39]. Those may be potential mechanisms. With higher platelet responsiveness and platelet counts, the platelet may release more pro-inflammatory factors and immune regulatory factors, and interact more with granulocytes, leading to more serious inflammatory reactions in female patients. In addition, a study reported that a higher MPV might be a surrogate marker associated with metabolic disturbance only in women [40], which may be another possible mechanism to explain our results. More researches are needed to furtherly confirm this relationship.

## Limitations

There were some limitations in this study. First, this is a single-center retrospective study with a small sample, so the results of this study ought to be generalized with caution. Second, we did not investigation nutritional status and antibiotics use which could affect the inflammatory response and in-hospital mortality. Third, there were about 20% postsurgical patients who tended to survive, this could cause lower in-hospital mortality compared to other studies [2, 5], and this possibly caused a bias in the interpretation of the results. Fourth, only the MPV within 24 h after intubation was studied, the change of MPV through ICU course were not evaluated due to lacking of data. Fifth, the AUC of 0.848 of MPV > 11.4 fl by ROC curve suggested that MPV alone is not enough to predict mortality. More predictive factors and models should be further studied in future. Sixth, the underlying mechanism of the sex difference between MPV and mortality was not clarified.

## Conclusion

The ability of MPV for predicting in-hospital mortality among patients undergoing IMV has not been studied before. The study suggested that MPV is a more useful maker for predicting in-hospital mortality, and MPV > 11.4 fl was an independent predictor factor for in-hospital mortality. This relationship was much stronger in female patients. MPV is reported in routine blood counts with low cost and availability, making it a profitable marker with immediate clinical applicability, especially in limited resource sites.

## Data Availability

The data sets supporting the results of this article are included within the article.

## Abbreviations

MPV: Mean platelet volume
IMV: Invasive mechanical ventilation
ICU: Intensive care unit
CRP: C-reaction protein
ROC: Receiver-operating characteristics
COPD: Chronic obstructive pulmonary disease
BMI: Body mass index
LOS: Length of stay
APACHE II: Acute physiology and chronic health evaluation II
RR: Respiratory rate
HR: Heart rate
SBP: Systolic blood pressure
SPO_2_: Peripheral oxygen saturation
PaCO2: Arterial carbon dioxide tension
PaO2: Arterial oxygen tension
FiO2: Fraction of inspired oxygen
MPV: Mean platelet volume
PDW: Platelet distribution width
ALT: Alanine aminotransferase
AST: Aspartate aminotransferase. AUC: area under the curve
CI: Confidence interval
HR: Hazard ratio
CRRT: Continuous renal replacement therapy
AKI: Acute kidney injury
CKD: Chronic kidney disease
